# Pilot Study of Embedded IMU Sensors and Machine Learning Algorithms for Automated Ice Hockey Stick Fitting

**DOI:** 10.3390/s22093419

**Published:** 2022-04-29

**Authors:** Taylor Léger, Philippe J. Renaud, Shawn M. Robbins, David J. Pearsall

**Affiliations:** 1 Department of Kinesiology and Physical Education, McGill Research Centre for Physical Activity and Health, McGill University, 475 Pine Avenue West, Montreal, QC H2W 1S4, Canada; taylor.leger@mail.mcgill.ca (T.L.); philippe.renaud@mcgill.ca (P.J.R.); 2 Centre for Interdisciplinary Research in Rehabilitation, Lethbridge-Layton-MacKay Rehabilitation Centre, the School of Physical and Occupational Therapy, McGill University, Montreal, QC H2W 1S4, Canada; shawn.robbins@mcgill.ca

**Keywords:** wearable sensors, ice hockey, hockey stick, equipment fitting, automation, machine learning

## Abstract

The aims of this study were to evaluate the feasibility of using IMU sensors and machine learning algorithms for the instantaneous fitting of ice hockey sticks. Ten experienced hockey players performed 80 shots using four sticks of differing constructions (i.e., each stick differed in stiffness, blade pattern, or kick point). Custom IMUs were embedded in a pair of hockey gloves to capture resultant linear acceleration and angular velocity of the hands during shooting while an 18-camera optical motion capture system and retroreflective markers were used to identify key shot events and measure puck speed, accuracy, and contact time with the stick blade. MATLAB R2020a’s Machine Learning Toolbox was used to build and evaluate the performance of machine learning algorithms using principal components of the resultant hand kinematic signals using principal components accounting for 95% of the variability and a five-fold cross validation. Fine k-nearest neighbors algorithms were found to be highly accurate, correctly classifying players by optimal stick flex, blade pattern, and kick point with 90–98% accuracy for slap shots and 93–97% accuracy for wrist shots in fractions of a second. Based on these findings, it appears promising that wearable sensors and machine learning algorithms can be used for reliable, rapid, and portable hockey stick fitting.

## 1. Introduction

Ice hockey sticks are a growing segment of the USD 850+ million hockey equipment market due to increasing global hockey participation and short product shelf life [1]. It is estimated that hockey sticks alone will reach a market value of USD 320 million by the end of 2025 [2]. Since players first began to modify their wooden sticks in the mid-20th century, the technologies, materials, and customizability of hockey sticks has improved greatly [3]. Today, players can purchase off the shelf sticks of various linear bending stiffnesses (“flex”), blade patterns (“curve” and “lie”), and dynamic flex profiles (“kick point”) with the rationale that different stick properties can enhance a player’s on-ice performance, such as shot speed, by “fitting” to their technique and/or style of play. During the shooting motion, the hockey stick functionally acts as a spring by releasing energy at a faster rate than it was previously stored, amplifying shot power.

Research on the influence of stick flex on shooting performance to date has been conflicted; however, it appears that players should select their flex based on the way they load the hockey stick. Three possible stick “loading styles” have been reported in the literature. These are Constant Displacement, Constant Force, and Constant Energy [4,5]. In each loading style, a player affects a constant maximal deformation of the shaft, applies an identical level of force, or stores the same maximal amount of energy in the stick regardless of changes in shaft stiffness. At present, the exact origins of these disparate stick loading styles remain unknown; however, there is evidence that such differences may arise due to differences in anthropometrics, kinematics, and levels of experience. For example, it is believed that larger, stronger players may be able to use a Constant Displacement model while weaker players may follow a Constant Force model [4]. Others have proposed that players have adopted new shooting techniques to take advantage of the spring-like properties of modern carbon composite hockey sticks [3]. These theories are consistent with previous findings that stick stiffness may negatively affect the shot velocities of younger [6] or female [7] players by preventing them from realizing the full power-amplifying benefits of the stick. These findings suggest that players should be provided tailored recommendations of shaft stiffness based on their specific loading style.

Sticks are designed to have specific kick points to maximize specific shot performance parameters, such as speed, accuracy, and quickness. Kick points are commonly referred to by the desired location of maximum deflection within the shaft. For example, a low-kick-point stick is most flexible at the bottom and increases in stiffness moving up the shaft while a high-kick-point stick is stiffest at the bottom. Published academic research on the effect of stick dynamic flex profiles is scarce. However, it is generally believed that low-kick-point sticks assist with release quickness while high-kick-point sticks are best for maximizing stick bend and puck velocity [8].

Research on the effect of blade patterns has shown that flatter curves may enhance shot speed by affecting the spatiotemporal characteristics of blade and stick loading during the slap shot [9]. This may occur due to systematically decreased lag between blade–toe and blade–heel ice contact similar to how advanced hockey players achieve higher puck speeds by “pinning” the stick blade earlier and storing more strain potential energy in the stick [10]. In contrast, the very origin of blade curves was to provide players with added levels of puck control [11]. Mechanically, more “open” curves make it easier to apply lift to the puck and consequently reach the upper net on forehand shots while a more pronounced curve and proper lie aid in puck control.

While hockey stick technologies have evolved drastically over the past twenty-five years, the means to fit players for them have been much slower to change; players still buy sticks mainly in retail stores without much knowledge of which may work best for them. The data-driven process of matching players with the best stick for them is called “stick fitting”. In recent years, with the incorporation of scanning technologies for hockey equipment fitting in retail settings, demand has grown for systems that are able to fit players quickly with little cost, floor space, or manpower required by the retailer. Previous research has shown that automated computer vision algorithms can evaluate shot performance metrics with comparable accuracy to more expensive and cumbersome motion capture systems [12]; however, the lengthy computation time (111 s/frame) makes it challenging to implement this automated, low-cost technology in retail settings. Instead, many hockey equipment retailers use 2D slow-motion video analysis apps, such as CCM Hockey’s Stick Fitter and Bauer Hockey’s Stick Studio, for stick fitting. No research has yet explored the combination of embedded sensors and machine learning algorithms for the purpose of automating stick fitting, providing recommendations of stick parameters more rapidly to players based on objective measures of shooting performance.

The aim of this study was to evaluate the feasibility of using inertial measurement unit sensors (IMUs) and machine learning algorithms for the stick fitting of experienced ice hockey players. This was an exploratory study on the use of in-glove IMUs in a hockey shooting context; however, motivation was derived from previous studies that have used kinematic signals obtained using IMUs and machine learning algorithms to classify athletic movement patterns [13,14], including those specific to ice hockey [15,16]. It was hypothesized that differences in hand kinematics would be characteristic of different responses to stick flex, blade pattern, and kick point.

## 2. Materials and Methods

### 2.1. Participants

Ten experienced male ice hockey players were recruited for this study. Two participants had experience at the Junior A level, five at the Canadian/American university level, and three at the professional level (Table 1). All participants had played hockey in the past calendar year and were free of serious injuries at the time of data collection. Descriptive statistics of the participants are provided in Table 1.

### 2.2. Instrumentation and Data Collection

Testing took place in the Biomechanics and Performance Analysis Lab at McGill University’s Currie Gymnasium in Montreal, Quebec. Testing procedures were explained in written and oral format to the participants, who then provided informed written consent prior to data collection.

An 18-camera Vicon optoelectronic system (Vicon, Oxford, UK) was used to capture three-dimensional kinematics of the body, stick, and puck during testing. These data were necessary to identify key shot events and calculate puck velocity (shot speed), radial error from target center at net entry (shot accuracy), and contact time with the stick blade (shot quickness). The formula of Hancock et al. [17] was used to calculate radial error, where x represents the horizontal distance between the puck center and target center and z represents the vertical distance between the puck center and target center at net entry:

(1) $$RE=\sqrt{\left(x^{2}+z^{2}\right)}.$$

**Figure 1:**
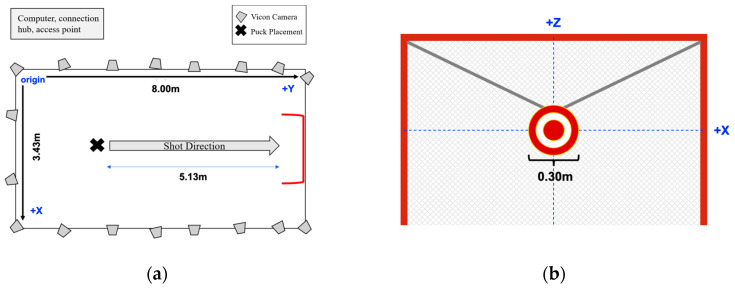
Schematic of the Biomechanics and Performance Analysis Lab layout: (**a**) top view of the motion capture area during data collection; and (**b**) net view, including the 0.30 m circular target—suspended in the center of the net using rope—which participants aimed for during each trial.

The Vicon system consisted of eight T10S, two T40S, four Vantage V5, and four Vero 2.2 cameras, which were positioned on tripods of varying heights around a synthetic ice surface (Viking, Toronto, ON, Canada) (Figure 1), providing a capture volume approximately 8.0 m long × 3.4 m wide × 2.0 m high. The cameras were connected to an MX Giganet connection Hub and desktop computer (Nexus 2.6, Vicon, Oxford, UK) and recorded at a sampling rate of 240 Hz.

**Figure 2:**
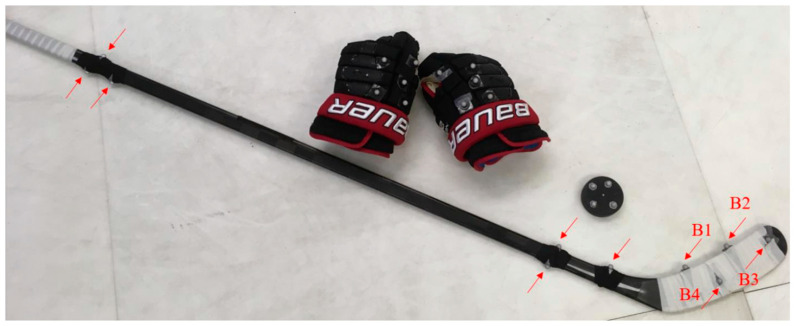
Reflective marker placement on gloves, stick, and puck for tracking by the Vicon system. B1–4 indicate markers used to define the movement of the stick blade during the shooting trials.

After obtaining informed written consent, anthropometric measurements were taken and participants were fitted with a full-body, tight-fitting Velcro suit (OptiTrack, Corvallis, OR, USA). Fifty-two spherical retroreflective markers with a diameter of 14 mm were placed on the participant in accordance with an adapted version of the Plug-in Gait full-body model (Figure 2). Four markers were placed on the puck and ten along each stick (Figure 2) to measure puck and stick kinematics, respectively. An additional eight markers were placed along the posts and crossbar of the net to compute puck-to-target accuracy measures.

Prior to the start of testing, participants recorded a static calibration pose held by each participant for five seconds with the arms parallel with the floor and elbow bent to 90°. This calibration was required to determine the model of each participant’s initial coordinate system reference frame. After the static calibration trial was recorded, participants were given time to warm-up and get accustomed to the synthetic ice surface by taking practice shots on the net.

Bespoke IMUs (Motsai Research, Saint-Bruno-de-Montarville, QC, Canada) were inserted in an opening in the thumb segment of each glove during testing to capture concurrent kinematics of the hands. This instrumentation configuration was chosen to ensure the IMU placement would not affect players’ shooting technique during data collection and could be easily replicated in a real-world fitting context. Data capture for the IMU system was initiated by a second researcher using a custom-built tablet application (Bauer Hockey Ltd., Blainville, QC, Canada) over Wi-Fi connection. The IMUs comprised an accelerometer capturing linear acceleration in gravitational units and a gyroscope capturing angular velocity in degrees per second at a rate of 200 Hz. Data collection was synchronized between the sensors and was triggered when either gyroscope signal passed a threshold of 4 degrees per second, recording for 199 frames.

### 2.3. Testing Protocol

Testing consisted of 10 stationary wrist shots and 10 stationary slap shots with 4 different sticks for a total of 80 shots. Stick A was the “baseline” stick, and Sticks B-D varied from Stick A in bending stiffness, blade pattern, or dynamic bending profile, respectively (Table 2). The sticks were all uniform in length and were representative of those commonly used by players of similar caliber. The sticks were devoid of graphics to blind participants to their differences. 

At the beginning of each trial, participants stood adjacent to the puck placed 5.13 m away from the net. Participants did not receive specific instructions with respect to their technique but were asked to shoot like they normally would in a game from a stationary position and to strive for maximum velocity and accuracy, aiming at a 0.3 m-diameter circular target suspended from the center of the net crossbar (Figure 1).

Each participant performed the first 20 trials with the reference stick (Stick A), with subsequent stick exposure orders (i.e., BCD, CDB, DBC, etc.) assigned using the first ten orders from a permutation generator to approximate a crossover-balanced design. Shot exposure order (wrist–slap–wrist... or slap–wrist–slap...) alternated between participants. Between each block of 10 shots, participants received approximately 1 min of rest.

### 2.4. Data Processing

Data captured using the Vicon cameras were processed using Vicon Nexus 2.6 software. Processing consisted of marker data identification (“labelling”) and gap filling (interpolating missing marker positions). This “processed” data from the Nexus software were then imported into Visual3D software (Ver 5.01.23, C-Motion, Germantown, MD, USA) where data were filtered using a 4th-order Butterworth filter with a cut-off frequency of 25 Hz, and all 3D calculations and event detections were performed. All data were reflected to one body side to account for kinematic differences due to handedness using the biomechZoo toolbox [17]. The terms “top” and “bottom” replaced “left” and “right” when identifying side-specific hand kinematic signals. Shot speed of each trial was calculated as peak puck velocity in the Y (towards the net) between puck contact/forward movement (START) and net entry (END) events for slap and wrist shots, respectively (Table 3).

**Figure 3:**
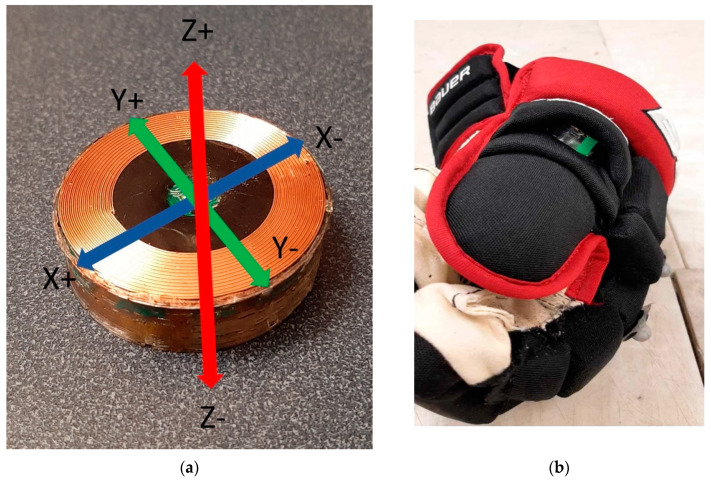
IMU axis alignment: (**a**) the +Z axis was orthogonal to the top surface of the IMU puck; and (**b**) the +Z was systematically aligned in the “thumbs up” direction of the glove; however, the direction of X and Y was not consistently maintained across testing.

For the IMUs, only the Z axis was systematically aligned in the glove due to technical challenges related to consistency. The +Z axis was aligned in the glove in the direction of thumb extension with the +X and +Y orthogonal to this axis (Figure 3). For this reason, only the resultant channels of the IMU’s accelerometer (linear acceleration in gravitational units) and gyroscope (angular velocity in degrees per second) were used for analysis. IMU data for both the left and right sensor were combined in the same file for each trial. Acceleration and gyroscope data were filtered with 4th-order lowpass Butterworth filter with a cut-off frequency of 20 Hz.

All trials were inspected for completeness, data quality, and event placement prior to the statistical analysis. Vicon trials were removed if certain events were unable to be detected in Visual3D; for example, if the trial ended prematurely or stick or puck markers had fallen off or went undetected by the Vicon system (*n* = 43). 

Since IMU trials did not have events, trials from this dataset were only omitted if they ended before the shooting motion had finished (*n* = 44). Due to differences in the causes of trial omission, the Vicon and IMU datasets were analyzed separately. Despite the fact they are of similar size, the datasets should not be confused as identical.

### 2.5. Model Training and Validation

Three separate models were created for each channel to fit players for flex, blade pattern, and kick point. Slap and wrist shot data were analyzed separately (3 stick parameters × 4 channels × 2 shot type = 24 models). The algorithms devised in this study were attempting to classify the responses of players’ shooting performance to stick flex, blade pattern, and kick point independently while each IMU channel provided different hand kinematic data throughout the shooting motion. Thus, the purpose of developing different sets of flex, blade pattern, and kick point models was to evaluate which IMU channel was best for each shot type and stick parameter combination.

Separate models were trained and tested on their ability to classify (i.e., fit) shooters using four different channels from the in-glove IMUs:

1. Top hand resultant linear acceleration (Top_A);
2. Top hand resultant angular velocity (Top_G);
3. Bottom hand resultant linear acceleration (Bottom_A);
4. Bottom hand resultant angular velocity (Bottom_G).

Three stick parameters were controlled in this study (flex, blade pattern, kick point). To determine a subject’s true class on each stick parameter, the shot speed of the stick that varied along that parameter (Stick B, Stick C, or Stick D, respectively) was compared to that of the reference stick (Stick A). True class values of stick fit—for training the algorithms and evaluating model accuracy—were based on the stick with higher mean shot speed to the nearest whole meter per second (m/s). For example, if a subject shot at least 1 m/s faster with Stick A (95 flex) than Stick B (70 flex), this subject would be considered a “high flex responder”. Shot speed was chosen as the shooting performance metric to define true class based on its prevalence in the literature [3,4,5,7,9,18,19] and its simplicity of measurement in various settings (e.g., using a radar gun).

The inputs of each model were reduced from 199 data points to the 14 to 18 principal components, or “features”, that explained 95% of the variability within the waveform data (Table 4). In other words, principal components—rather than complete waveforms—were the features used to develop the algorithms. The features, in addition to true class values, were fed into MATLAB R2020a’s Machine Learning Toolbox, and prediction models were created using k-nearest neighbors (KNN) classification. Five-fold cross validation—such as the principal components—was used to improve the generalizability of the models.

For all models, standardized data, a Euclidean distance metric, equal distance weight, and a default misclassification cost matrix were used, and hyper-parametrization was disabled.

Model performance was evaluated primarily on classification accuracy (high being preferable) and secondarily on computation time (low being preferable) after the fifth fold of cross-validation was used to compare performance between models.

## 3. Results

### 3.1. Shooting Performance Variables

**Figure 4:**
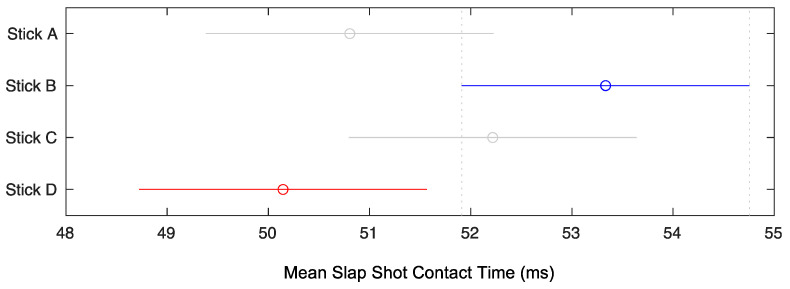
Mean slap shot blade–puck contact time (ms) ± standard error. The means of Stick B and Stick D are significantly different.

One-way ANOVAs revealed significant differences (α < 0.05) in slap shot blade contact time between sticks (*F*(3.36) = 3.66, *p* = 0.021, η_p_^2^ = 0.234), but not for slap shot speed (*F*(3.36) = 0.032, *p* = 0.992, η_p_^2^ = 0.002) nor accuracy (*F*(3.36) = 2.33, *p* = 0.091, η_p_^2^ = 0.162) (Table 5). A Tukey–Kramer post hoc test showed contact time to be significantly shorter for Stick D compared to Stick B (Figure 4). 

**Figure 5:**
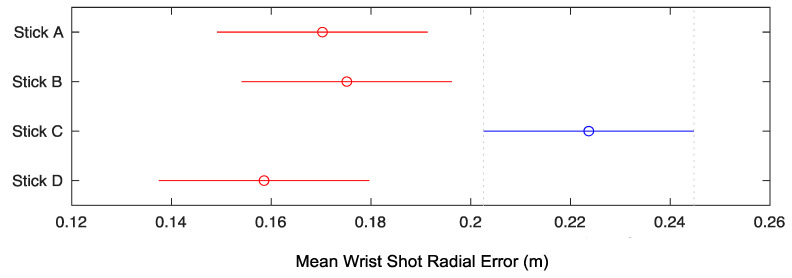
Mean wrist shot radial error from target center (m) ± standard error. The mean of Stick C is significantly different from Sticks A, B, and D.

For wrist shots, one-way ANOVAs revealed significant differences in accuracy between sticks (*F*(3.36) = 6.71, *p* = 0.001, η_p_^2^ = 0.359). A Tukey–Kramer post hoc test showed wrist shot accuracy to be significantly lower for Stick C—by means of a higher average radial error—than the other three sticks (Figure 5).

### 3.2. Machine Learning Model Accuracy

All of the most successful models were nearest neighbor algorithms (i.e., k = 1). Thus, in the interest of space, only the performances of these models are presented.

#### 3.2.1. Slap Shots

Based on the models created, principal components of the bottom hand angular velocity (Bottom_G) data were able to recommend the optimal flex (96%), blade pattern (98%), and kick point (97%) for slap shot speed with the highest accuracy (Table 6).

#### 3.2.2. Wrist Shots

Based on the models created, principal components of the top hand angular velocity (Top_G) data were able to recommend the optimal kick point (97%) for wrist shot speed with the greatest accuracy, while principal components of bottom hand linear acceleration (Bottom_A) best predicted optimal flex (97%) and blade pattern (96%) (Table 7).

### 3.3. Classification Confusion Matrices

#### 3.3.1. Slap Shots

**Figure 6:**
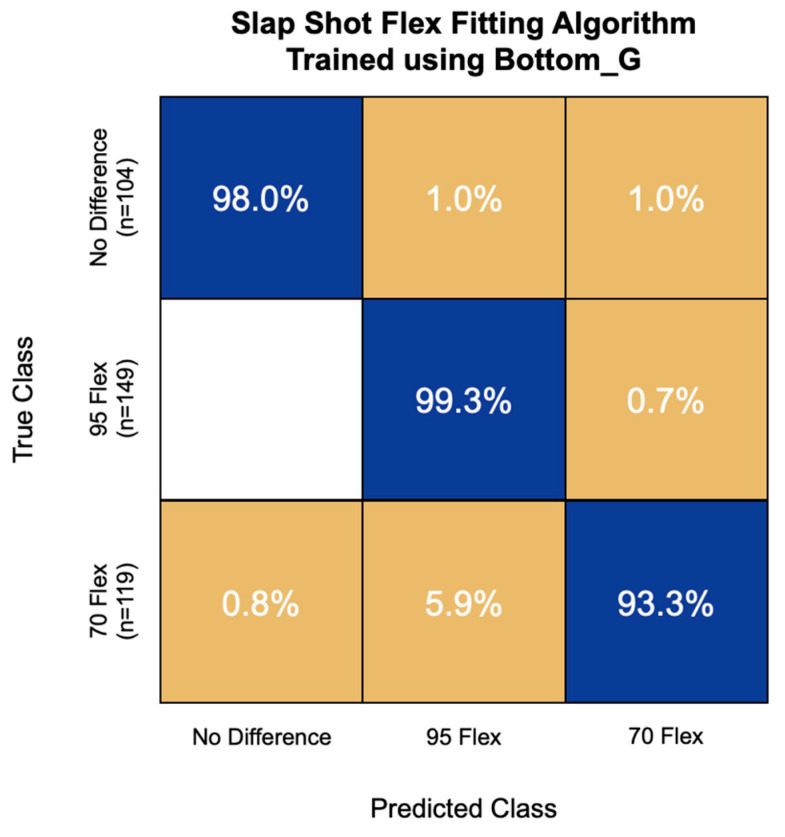
Confusion matrix of the flex fitting algorithm for slap shot speed trained using bottom hand resultant angular velocity (Bottom_G) signal features. Overall model accuracy was 97.3%.

The most accurate flex fitting algorithm for slap shot speed used the first 15 principal components of the Bottom_G IMU channel. This model had an overall accuracy of 97.3%, correctly classifying 98.0% of non-responders, 99.3% of high-flex responders, and 93.3% of low-flex responders (Figure 6). This model never misclassified a high-flex responder for a non-responder and had the greatest challenge with low-flex responders, misclassifying 5.9% of them as high-flex responders. 

**Figure 7:**
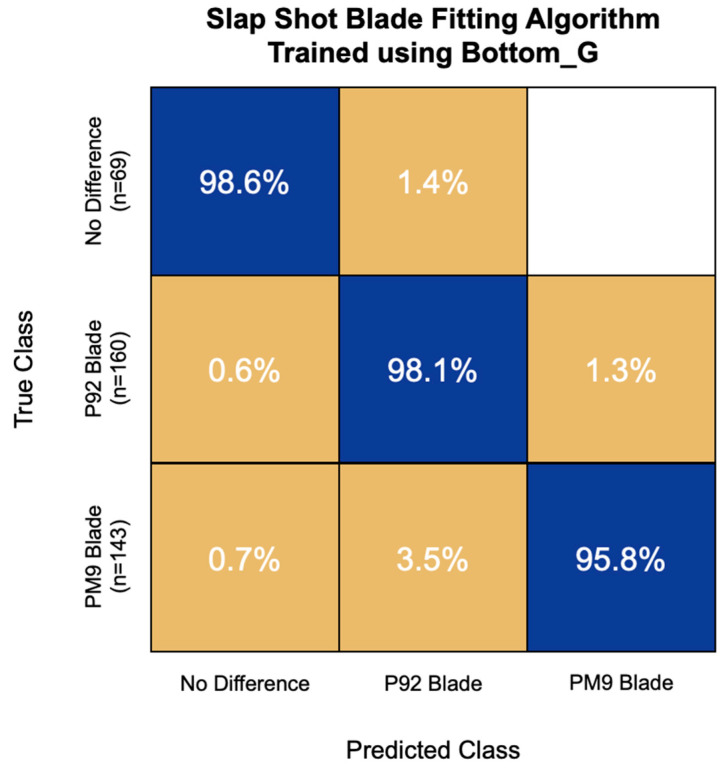
Confusion matrix of the blade fitting algorithm for slap shot speed trained using bottom hand resultant angular velocity (Bottom_G) signal features. Overall model accuracy was 97.3%.

The most accurate blade fitting algorithm for slap shot speed was trained using the first 15 principal components of the Bottom_G IMU channel. This model also had an overall accuracy of 97.3% and correctly classified 98.6% of non-responders, 98.1% of P92 responders, and 95.8% of PM9 responders (Figure 7). This model never misclassified a non-responder for a PM9 responder and had the greatest challenge with PM9 responders, misclassifying 3.5% of them as P92 responders.

**Figure 8:**
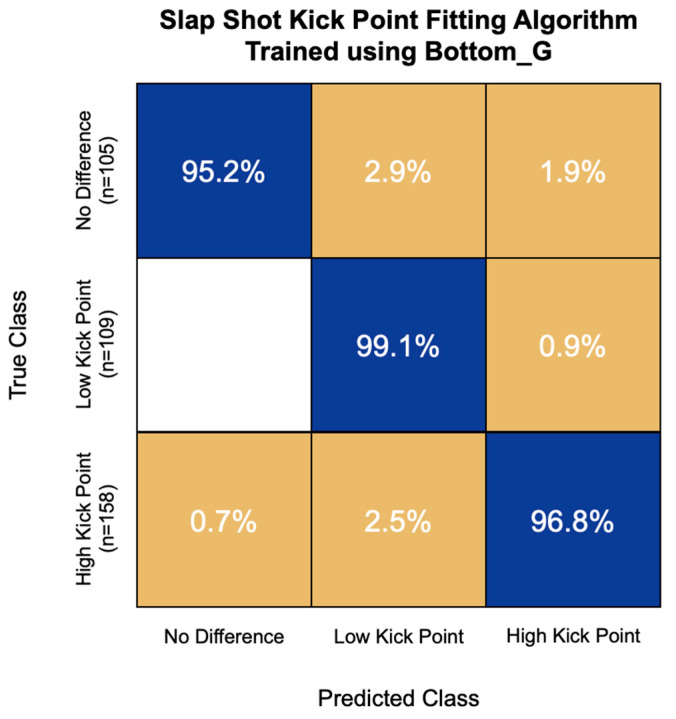
Confusion matrix of the kick point fitting algorithm for slap shot speed trained using bottom hand resultant angular velocity (Bottom_G) signal features. Overall model accuracy was 97.0%.

The highest accuracy kick point fitting algorithm for slap shot speed was also trained using the first 15 principal components of the Bottom_G IMU channel. This model had an overall accuracy of 97.0% and correctly classified 95.2% of non-responders, 99.1% of low-kick-point responders, and 96.8% of high-kick-point responders (Figure 8). This model never misclassified a low-kick-point responder for a non-responder and had the greatest challenge with high-kick-point responders, misclassifying 2.5% of them as low-kick-point responders.

#### 3.3.2. Wrist Shots

**Figure 9:**
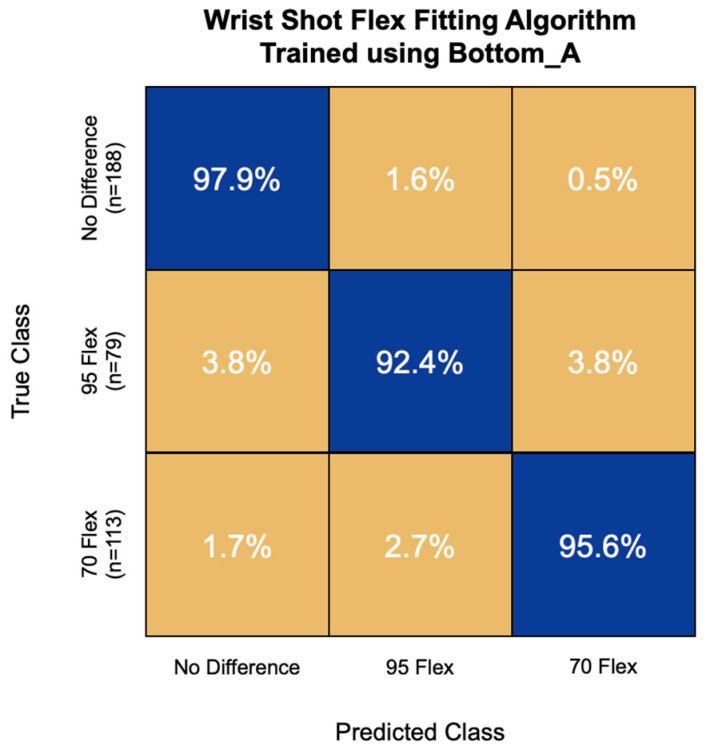
Confusion matrix of the flex fitting algorithm for wrist shot speed trained using bottom hand resultant linear acceleration (Bottom_A) signal features. Overall model accuracy was 96.1%.

The highest accuracy flex fitting algorithm for wrist shot speed was trained using the first 16 principal components of the Bottom_A IMU channel. This model had an overall accuracy of 96.1% and correctly classified 97.9% of non-responders, 92.4% of high-flex responders, and 95.6% of low-flex responders (Figure 9). 

**Figure 10:**
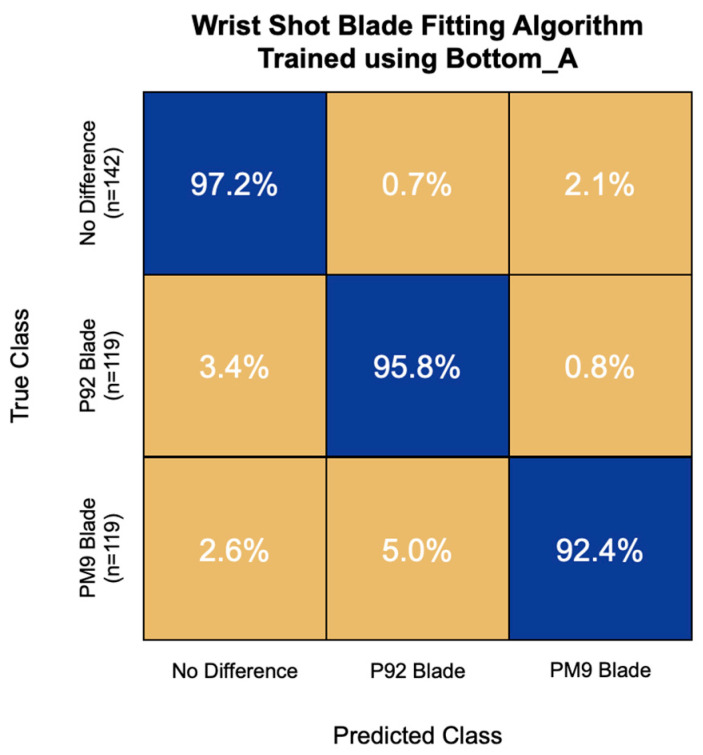
Confusion matrix of the blade fitting algorithm for wrist shot speed trained using bottom hand linear acceleration (Bottom_A) signal features. Overall model accuracy was 95.3%.

In addition, the most accurate blade fitting algorithm for wrist shot speed was trained using the first 16 principal components of the Bottom_A IMU channel. This model had an overall accuracy of 95.3% and correctly classified 97.2% of non-responders, 95.8% of P92 responders, and 92.4% of PM9 responders (Figure 10). This model had the greatest challenge with PM9 responders, misclassifying 5.0% of them as P92 responders.

**Figure 11:**
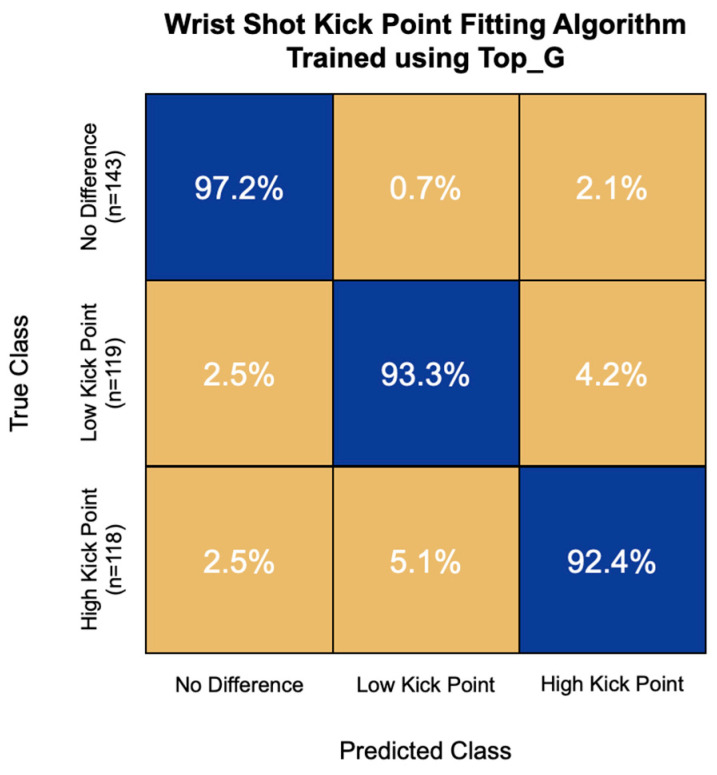
Confusion matrix of the kick point fitting algorithm for wrist shot speed trained using top hand resultant angular velocity (Top_G) signal features. Overall model accuracy was 94.5%.

The highest accuracy kick point fitting algorithm for wrist shot speed was trained using the first 15 principal components of the Top_G IMU channel. This model had an accuracy of 94.5% and correctly classified 97.2% of non-responders, 93.3% of low-kick-point responders, and 92.4% of high-kick-point responders (Figure 11). This model had the greatest challenge with high-kick-point responders, misclassifying 5.1% of them as low-kick-point responders.

### 3.4. Computation Time and Misclassification Costs

A key factor of interest in the evaluation of these machine learning algorithms was computation time. The prediction speeds of the best models, in addition to misclassification costs for each algorithm, are presented in Table 8. These include the Bottom_G models for fitting flex, blade, and kick point for slap shots and the Bottom_A, Bottom_A, and Top_G models for fitting wrist shot flex, blade, and kick point, respectively. All computation times were less than one second.

## 4. Discussion

Using in-glove IMU data, fine KNN machine learning models were capable of fitting players with their optimal flex, blade pattern, and kick point with 90–98% accuracy for slap shots and 93–97% accuracy for wrist shots. Perhaps more impressively, these algorithms were able to achieve these high levels of accuracy with a processing time of less than one second. Thus, it appears possible—using embedded sensors and fine KNN machine learning algorithms—to rapidly fit players for stick flex, blade, and kick point with high levels of accuracy.

Individual differences in optimal sticks for shot speed reported corroborate trends in previous hockey stick studies [11] and those of other sporting implements [20]. Anderson found three players using six different sticks of wood and composite constructions each shot fastest with a different stick.

More specifically, machine learning algorithms were able to identify individual differences in hand kinematic signals to predict optimal stick flex, blade pattern, and kick point based on shot velocity. This agrees with previous findings of Zane [21] and Flemming [22], who reported player coordinative strategies (or “shooting styles”) that appeared in both high- and low-caliber groups. It has been established that no two players shoot exactly the same [22]; however, the methods used in this study were satisfactorily sensitive to the characteristics of hand kinematics relevant to stick fitting for the purpose of maximizing shot speed. Thus, further research is warranted to better understand how features of these kinematic profiles relate to stick fit and employ this technology to assist players in tuning the flex, blade pattern, and kick point that they use. 

The small sample size (*n* = 10) may have limited the statistical power of the analysis. A larger sample size (e.g., *n* = 40) of novel hockey players of differing ability levels would better permit the comparison of different machine learning algorithms in predicting hockey stick preferences in future studies [14,23]. Further, it is unknown how the findings of the present study apply to the larger population of hockey players, since it is not known how representative this sample is of the kinematic variability across different player demographics. For example, only experienced adult males participated in this study. Thus, the inclusion of female, youth, and less experienced athletes for the development of commercial hockey stick fitting applications is warranted since these groups represent large, growing segments of the global hockey stick market [24].

Another limitation of this study was that the IMU data were limited to the resultant magnitude of the accelerometer and gyroscope data due to the lack of systematic alignment of the IMUs’ X and Y axes in the gloves. Nonetheless, the IMUs provided unencumbered, direct kinematic measurements of the hands, which makes them practical for application in stick fitting in retail hockey stores. More work is required to investigate the repeatability of these findings when accounting for IMU alignment.

Moreover, the current algorithm was delimited to the fitting of sticks based on shot velocity; however, shot accuracy and quickness may be of greater importance to a player depending on their position and style of play [3]. Thus, it is suggested that future stick fitting research considers the contribution of such metrics to shooting performance at large. This could elucidate additional clusters of hockey shooters. IMU alignment, as mentioned above, would assist in ensuring the reliability of such algorithms.

An additional limitation of this study was that the sticks were not subjected to a battery of mechanical tests to understand the physical properties of sticks [25]. Having such information would be interesting, particularly for understanding how such parameters relate to perceptions of shooting performance [26,27]. Further, understanding how perceptual and performance measures relate to dynamic player behaviors during shooting—such as grip width on the stick and stick bend—is of great interest.

In summary, these findings suggest that there are promising applications for the combination of embedded IMU sensors and machine learning algorithms in the fitting of ice hockey sticks due to the portability, accuracy, and processing speed of such systems. This technology could be used to recommend hockey stick parameters quickly, accurately, and scientifically in a format easily accessible to hockey players. In the future, standalone IMU systems could be used to further differentiate hockey players based on their shooting kinematics and provide more individualized fitting recommendations. Further research to investigate the repeatability of these findings within a larger, diverse, and representative group of hockey players is warranted.

## 5. Conclusions

Fine k-nearest neighbors machine learning models were capable of fitting players with their optimal flex, blade pattern, and kick point with 90–98% accuracy for slap shots and 93–97% accuracy for wrist shots using select principal components of in-glove IMU data. Perhaps more impressively, these algorithms were able to achieve these high levels of accuracy with a processing time of less than one second.

The above results demonstrate the potential of using embedded IMU sensors tracking hand kinematics and machine learning algorithms to accurately fit individuals for stick flex, blade pattern, and kick point. Similar research to this study should be conducted with a greater number of sticks with more subtle differences in flex, blade patterns, and kick points and a larger pool of hockey players of differing backgrounds to further validate the findings of this study. This study could be further supplemented through the capture of shooting performance and stick perception data in retail and on-ice settings using simplified objective measurement instruments (i.e., a radar gun).

Although it appears that players do not change their shooting style when using sticks of different flexes in the short run, it is not known how stable these gross kinematic patterns are and whether they would yield consistent values under test–retest conditions. Additionally, it is not yet known how habitual exposure to sticks of different flex, blade, and kick point properties affect shooting technique. Studies aimed at the repeat exposure and measurement of kinematics in response to sticks with different properties should be conducted.

## Figures and Tables

**Table 1:** Descriptive statistics of study participants.

Measure	Mean ± SD
Age (yrs)	25.9 ± 4.9
Height (m)	1.81 ± 0.07
Body Mass (kg)	88.5 ± 7.7
Hockey Playing Experience (yrs)	20.3 ± 3.7

**Table 2:** Specifications of the four sticks used during testing. Stick A was used as a “baseline” implement. Stick B varied in stiffness. Stick C varied in blade pattern. Stick D varied in kick point.

Stick	Stiffness Rating	Blade Pattern	Kick Point
Stick A	95	P92	Low
Stick B	70	P92	Low
Stick C	95	PM9	Low
Stick D	95	P92	High

**Table 3:** Definitions of shot events used to calculate shooting performance metrics with the Vicon system.

Shot Type	Event	Description
Slap	START	One frame before puck velocity in the Y exceeded 5.0 m/s (i.e., when the blade made contact with the puck).
	END	The frame when the distance between the puck and the net was less than 0 m in the Y (i.e., when the puck “entered” the net).
Wrist	START	The frame when blade acceleration in the Y exceeded 150 m/s^2^ (i.e., when the blade begins moving forward).
	END	The frame when the distance between the puck and the net was less than 0 m in the Y (i.e., when the puck “entered” the net).

**Table 4:** Number of trials (*n*) and features (PC) used for each algorithm.

Shot Type	Characteristic	Top_A	Top_G	Bottom_A	Bottom_G
Slap	*n*	374	374	372	372
	*PC*	18	14	16	15
Wrist	*n*	380	380	380	380
	*PC*	15	15	16	15

**Table 5:** Ensemble average speed, accuracy, and release quickness metrics with each stick for slap and wrist shots ± SD (*n* = 10).

Shot Type	Performance Metric	Stick A	Stick B	Stick C	Stick D
Slap	Speed (m/s)	31.7 ± 2.2	31.8 ± 1.9	31.9 ± 1.7	32.0 ± 1.8
	Radial Error (m)	0.296 ± 0.166	0.282 ± 0.138	0.321 ± 0.157	0.224 ± 0.129
	Contact Time (ms)	51 ± 4	53 ± 5	52 ± 4	50 ± 4
Wrist	Speed (m/s)	27.7 ± 1.6	27.9 ± 1.4	27.6 ± 1.5	27.4 ± 1.4
	Radial Error (m)	0.171 ± 0.089	0.177 ± 0.089	0.224 ± 0.138	0.159 ± 0.090
	Contact Time (ms)	103 ± 12	107 ± 13	104 ± 12	104 ± 13

**Table 6:** Accuracy of various fine KNN models trained using top and bottom hand linear acceleration (_A) and angular velocity (_G) data for slap shot speed.

Fit Characteristic	Top_A	Top_G	Bottom_A	Bottom_G
Flex	95%	95%	93%	96%
Blade	95%	95%	90%	98%
Kick Point	93%	96%	94%	97%

**Table 7:** Accuracy of various fine KNN models trained using top and bottom hand linear acceleration (_A) and angular velocity (_G) data for wrist shot speed.

Fit Characteristic	Top_A	Top_G	Bottom_A	Bottom_G
Flex	93%	95%	97%	96%
Blade	93%	93%	96%	95%
Kick Point	94%	97%	96%	94%

**Table 8:** Total misclassification cost and prediction speed of each of the six most accurate models.

Shot Type	Model	Total Misclassification Cost	Observations/Second
Slap	Flex	10	~700
	Blade	10	~1200
	Kick Point	11	~1700
Wrist	Flex	15	~1400
	Blade	18	~1400
	Kick Point	21	~1400

## Data Availability

The data presented in this study are available on request from the corresponding author. The data are not publicly available due to participant privacy.

## References

[1] (2019). Grand View Research Ice Hockey Equipment Market Size, Share & Trends Analysis Report by Product Type (Protective Wear, Sticks), by Distribution Channel (Online, Offline), by Region, and Segment Forecasts, 2019–2025.

[2] More A. (2019). Ice Hockey Stick Market Share, Size 2019—Global Industry Future Demand, Global Research, Top Leading Players, Emerging Trends, Region by Forecast to 2025.

[3] Pearsall D., Robbins S., Subic A. (2019). Design and Materials in Ice Hockey. Materials in Sports Equipment.

[4] Kays B.T., Smith L.V. (2017). Effect of Ice Hockey Stick Stiffness on Performance. Sports Eng..

[5] Worobets J.T., Fairbairn J.C., Stefanyshyn D.J. (2006). The Influence of Shaft Stiffness on Potential Energy and Puck Speed during Wrist and Slap Shots in Ice Hockey. Sports Eng..

[6] Roy B., Doré R. (1976). Kinematics of the Slap Shot in Ice Hockey as Executed by Players of Different Age Classifications. Biomechanics V-B: Proceedings of the Fifth International Congress on Biomechanics.

[7] Gilenstam K., Henriksson-Larsén K., Thorsen K. (2009). Influence of Stick Stiffness and Puck Weight on Puck Velocity during Slap Shots in Women’s Ice Hockey. Sports Eng..

[8] Legault M. Advanced Hockey Stick Design Delivers Optimal Performance. https://www.compositesworld.com/articles/advanced-hockey-stick-design-delivers-optimal-performance.

[9] Gerbé A. (2016). Analysis of Hockey Blade Dynamic Behavior Using Digital Image Correlation (DIC). Master’s Thesis.

[10] Lomond K.V., Turcotte R.A., Pearsall D.J. (2007). Three-Dimensional Analysis of Blade Contact in an Ice Hockey Slap Shot, in Relation to Player Skill. Sports Eng..

[11] Anderson R.L. (2008). Experimental Characterization of Ice Hockey Sticks and Pucks. Master’s Thesis.

[12] Mendhurwar K., Handa G., Zhu L., Mudur S., Beauchesne E., LeVangie M., Hallihan A., Javadtalab A., Popa T. A System for Acquisition and Modelling of Ice-Hockey Stick Shape Deformation from Player Shot Videos. Proceedings of the 2020 IEEE/CVF Conference on Computer Vision and Pattern Recognition Workshops (CVPRW).

[13] Kim M., Park S. (2020). Golf Swing Segmentation from a Single IMU Using Machine Learning. Sensors.

[14] McGrath J., Neville J., Stewart T., Clinning H., Cronin J. (2021). Can an Inertial Measurement Unit (IMU) in Combination with Machine Learning Measure Fast Bowling Speed and Perceived Intensity in Cricket?. J. Sports Sci..

[15] Gal A.M., Douglas T., Chan A.D.C., Hay D.C. Potential for Autonomous Linear, Curvilinear, and Phase Detection in Natural Context Para Skating Using IMU Sledge Motion Data. Proceedings of the 2020 IEEE International Symposium on Medical Measurements and Applications (MeMeA).

[16] Vleugels R., Van Herbruggen B., Fontaine J., De Poorter E. (2021). Ultra-Wideband Indoor Positioning and IMU-Based Activity Recognition for Ice Hockey Analytics. Sensors.

[17] Dixon P.C. (2017). BiomechZoo: An Open-Source Toolbox for the Processing, Analysis, and Visualization of Biomechanical Movement Data. Comput. Methods Programs Biomed..

[18] Alexander J.F., Haddow J.B., Schultz G.A. (1963). Comparison of the Ice Hockey Wrist and Slap Shots for Speed and Accuracy. Res. Q. Am. Assoc. Health Phys. Educ. Recreat..

[19] Robbins S.M., Renaud P., MacInnis N., Pearsall D. (2021). The Relationship between Trunk Rotation and Shot Speed When Performing Ice Hockey Wrist Shots. J. Sports Sci..

[20] MacKenzie S.J., Boucher D.E. (2017). The Influence of Golf Shaft Stiffness on Grip and Clubhead Kinematics. J. Sports Sci..

[21] Zane L. (2012). Force Measures at the Hand-Stick Interface during Ice Hockey Slap and Wrist Shots. Master’s Thesis.

[22] Flemming A. (2014). The Dynamic Interaction of Hand Grip and Ice Hockey Stick Flexion during Slap Shots and Wrist Shots. Master’s Thesis.

[23] Balsalobre-Fernández C., Kipp K. (2021). Use of Machine-Learning and Load–Velocity Profiling to Estimate 1-Repetition Maximums for Two Variations of the Bench-Press Exercise. Sports.

[24] (2020). Hockey Canada Annual Report: July 2019–June 2020.

[25] Behrmann L., Litzenberger S., Mally F., Sabo A. (2014). Evaluation of Bending and Torsional Properties of Different Ice Hockey Sticks. Procedia Eng..

[26] Fischer C., Overney L.S., Fauve M., Blanke O., Rhyner H., Herzog M.H., Bourban P.-E., Månson J.-A.E. (2007). What Static and Dynamic Properties Should Slalom Skis Possess? Judgements by Advanced and Expert Skiers. J. Sports Sci..

[27] Overney L.S., Michaud V., Fischer C., Heubi J., Veldhuis L., Blanke O., Herzog M.H., Månson J.-A. (2010). Carbon Outclasses Wood in Racket Paddles: Ratings of Expert and Intermediate Tennis Players. J. Sports Sci..

